# Rhein Inhibits the Progression of Chemoresistant Lung Cancer Cell Lines via the Stat3/Snail/MMP2/MMP9 Pathway

**DOI:** 10.1155/2022/7184871

**Published:** 2022-02-08

**Authors:** Jinxin Liu, Dapeng Ding, Feiye Liu, Yizhi Chen

**Affiliations:** ^1^Department of Oncology, Longgang District Central Hospital of Shenzhen, Shenzhen 518116, China; ^2^Department of Biochemistry and Molecular Biology, School of Basic Medical Sciences, Southern Medical University, Guangzhou 510515, China; ^3^Department of Oncology, Traditional Chinese Medicine-Integrated Hospital, Southern Medical University, Guangzhou 510315, China; ^4^Department of Health Records, Longgang District Central Hospital of Shenzhen, Shenzhen 518116, China

## Abstract

Chemotherapy is a common drug for lung cancer. Nevertheless, the development of drug resistance greatly limits their clinical efficacy. Therefore, to reduce drug resistance, we need to constantly explore new treatments. This study is aimed at determining the role of rhein in the proliferation and metastasis of lung cancer cell. Our study found that rhein significantly inhibits the proliferation and migration of lung cancer cells. Additionally, the mRNA expression and protein levels of Snail, MMP2, and MMP9 are decreasing in lung cancer cells treated by rhein. Our results showed that rhein plays a vital role in proliferation and metastasis of chemosensitive and chemoresistant lung cancer cells, and the mechanism may be related to the Stat3/Snail/MMP2/MMP9 pathway.

## 1. Introduction

Lung cancer is one of the most common tumors (11.6% of all cases) and accounts for a higher proportion of tumor deaths (18.4% of all cancer deaths) [1]. The main treatments for lung cancer are still surgery, radiotherapy, and chemotherapy. However, most lung cancer patients are in the advanced stages of the disease, and surgical treatment is no longer the best option. Therefore, chemotherapy and targeted therapies have become the first choice of treatment [2].

In recent years, due to the continuous optimization of treatment regiments and the use of targeted drugs, the treatment effect of lung cancer has been significantly improved. Gefitinib and Taxol are considered as effective drugs in the treatment of lung cancer [3–5]. Nevertheless, the development of drug resistance greatly limits their clinical efficacy [6, 7]. Therefore, in order to improve the quality of life and treatment effect of lung cancer patients, we should explore new therapeutic therapies.

Traditional Chinese medicine preparation has become a breakthrough point in the research of antitumor mechanism because of its multitarget and high curative effect. Rhein is one of the effective components extracted from Polygonaceae plants [8]. A large number of literatures have reported that rhein plays an antitumor role by inducing tumor cell apoptosis, affecting tumor cell proliferation, and inhibiting tumor cell migration and invasion in breast cancer, colon cancer, and lung cancer cells [9–16]. However, the mechanism of its action on proliferation and metastasis and the signaling pathways involved are still unclear.

Matrix metalloproteinases (MMPs) are a group of zinc-dependent metalloenzymes that regulate a variety of cellular processes, including tumor cell proliferation and metastasis [17]. MMP2 and MMP9 are two special subtypes of MMPs, which have been deeply studied in cancer metastasis in recent years [18–20]. Snail is a typical member of the epithelial-mesenchymal cell transition-induced transcription (EMT) factor family [21]. Snail can enhance invasiveness and metastasis [22, 23]. Signal transducer and activator of transcription-3 (Stat3) is a potential target for many cancers, including lung cancer. Stat3 has the effect of inhibiting apoptosis and promoting cell proliferation during tumorigenesis [24–27]. Moreover, Stat3 can activate the transcription of MMP2, MMP9, and Snail in cancer cells [28, 29]. Therefore, inhibiting Stat3 activation may be an effective way to treat lung cancer.

In this study, our results showed that rhein have obviously affect in treatment of proliferation and metastasis of chemosensitive and chemoresistant lung cancer cells, and play a role via the Stat3/Snail/MMP2/MMP9 pathway.

## 2. Materials and Methods

### 2.1. Materials and Reagents

RPMI-1640 medium, penicillin-streptomycin, 0.25% trypsin-EDTA, fetal bovine serum (FBS), and TRIzol were purchased from Thermo Fisher Scientific (Waltham, MA, USA). 3-[4,5-Dimethylthiazol-2-yl]-2,5-diphenyltetrazolium bromide (MTT) was purchased from Solarbio Science & Technology (Beijing, China). iScript^™^ cDNA Synthesis Kit and SsoFast EvaGreen Supermix were purchased from Bio-Rad Laboratories (Hercules, CA). MMP2 (10373-2-AP), MMP9 (10375-2-AP), *β*-actin (20536-1-AP), and STAT3 (10253-2-AP) were purchased from Proteintech (Wuhan, China). Rabbit polyclonal antibodies against Phospho-Stat3 (9145S) and Snail (3879S) were purchased from Cell Signaling Technology (Dallas, Texas, USA). Horseradish peroxidase- (HRP-) conjugated goat anti-rabbit (E030120-01) was purchased from EarthOx (Danvers, MA, USA). Culture plates and flask were purchased from Corning (New York, USA). Rhein and all the other chemicals used, unless otherwise stated, were purchased from Sigma Chemicals (St. Louis, MO, USA).

### 2.2. Cell Lines and Culture

We bought human lung cancer cells (A549, A549/Taxol, PC9, and PC9/GD) from the Cell Institute of Chinese Academy of Sciences. Cell culture is carried out in a cell culture box, culture is the addition of antibiotics (100 U/ml penicillin and 100 *μ*g/ml streptomycin), and the culture base is RPMI1640 containing 10% fetal bovine serum. The conditions for cell culture are 37°C humidified incubator with 5% CO_2_.

### 2.3. Cell Viability Assay

3-(4,5)-Dimethylthiahiazo (-z-y1)-3,5-di-phenytetrazoliumromide (MTT) assay was used to assess the cell viability. A549, A549/Taxol, PC9, and PC9/GD cells were cultured into 96-well plates. The cell concentration is 1 × 10^4^ cells/ml in 200 *μ*l culture medium for 12 hours. The cells are then stimulated with different concentrations of Taxol, gefitinib, or rhein for 24 h. Afterwards, 2 *μ*l of MTT (5 mg/ml in PBS) was added into the culture medium and the samples were incubated for an additional 4 h at 37°C. The MTT formazan precipitate was dissolved in 150 *μ*l DMSO. The resulting absorbance was measured at 490 nm.

### 2.4. Colony Formation

A549, A549/Taxol, PC9, and PC9/GD cells were seeded into 6-well plates at a density of 3 × 10^5^ cells/ml in 2 ml culture medium. The detailed experimental process refers to the previous studies [10].

### 2.5. Wound Healing Assay

The detailed experimental process refers to the previous studies [10]. Briefly, the cells were seeded into 6-well plates, at 80–90% confluence; a 200 *μ*l pipette tip was used to scrape the cells. Afterward, the cells were further incubated with 25 *μ*g/ml or 50 *μ*g/ml rhein for 24 h in a serum-free RPMI 1640 to examine cell migration in the absence of cell growth. Cell migration was detected by a phase-contrast microscope at 0 and 48 h. The ImageJ software was used to measure and quantify the denuded area.

### 2.6. Transwell Migration Assay

Cell migration was analyzed in Transwell chambers (8 *μ*m pore size, BD Biosciences, NJ, USA). The detailed experimental process refers to previous studies [10]. After 24 h cell culture, the membranes were fixed in 4% paraformaldehyde/PBS for 10 min and 0.1% crystal violet was used to stain the cells on the underside of cell membrane. The cells on the underside of Transwell membrane were measured and counted by a microscope (100× magnification).

### 2.7. Real-Time Polymerase Chain Reaction (PCR)

The cells were harvested separately after rhein treatment at 25 *μ*g/ml or 50 *μ*g/ml for 24 h. Total RNA was isolated from cells using a TRIzol and reverse transcribed to form single-strand cDNA with an iScript^™^ cDNA Synthesis Kit. Real-time PCR was conducted using an Applied Biosystems^™^ 7500 Real-Time PCR System and SsoFast EvaGreen Supermix. *β*-Actin served as the endogenous control. Relative expression was calculated and normalized to *β*-actin using the 2^−*ΔΔ*Ct^ methods.

### 2.8. Western Blot Analysis

The cells were challenged with 25 *μ*g/ml or 50 *μ*g/ml rhein for 24 h. Afterwards, cells were rinsed with PBS buffer for three times and lysed with RIPA lysis buffer. The total proteins were extracted from the cells and resolved through 15% SDS-PAGE and then transferred onto polyvinylidene fluoride (PVDF) membrane. After incubation with the specific primary and secondary antibody, protein expressions were detected by ECL kit. ImageJ was used for densitometric analysis.

### 2.9. Statistical Analysis

SPSS for Windows (version 22.0, SPSS Inc., Chicago, IL, USA) was used in this study. Statistical analysis of the data was performed using one-way analysis of variance with post hoc test of least significant difference (LSD). *P* < 0.05 was considered statistically significant.

## 3. Results

### 3.1. Rhein Suppresses Cell Proliferation in Chemosensitive and Chemoresistant Lung Cancer Cells

**Figure 1:**
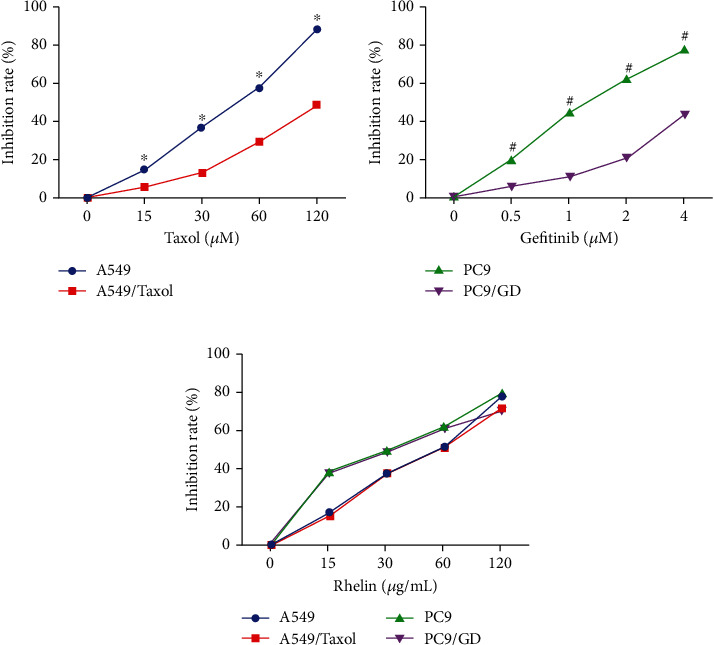
Rhein suppresses cell proliferation in chemosensitive and chemoresistant lung cancer cells. (a) Inhibition of A549 and A549/Taxol cell survival treated with Taxol (0, 15, 30, 60, and 120 *μ*M). (b) Inhibition of PC9 and PC9/GD cell survival treated with gefitinib (0, 0.5, 1, 2, and 4 *μ*M). (c) Inhibition of A549, A549/Taxol, PC9, and PC9/GD cell survival treated with rhein (0, 5, 25, 50, and 100 *μ*M) for 24 h. The inhibition rate was calculated in comparison to control cells. The number of cells in the control group was taken as 100%. Data are from three independent experiments. Results are shown as the means ± SD (*n* = 3). ^∗^*P* < 0.05 compared with A549/Taxol and ^#^*P* < 0.05 PC9/GD group treated with the same dose of Taxol, gefitinib, and rhein, respectively.

In order to evaluate the effect of rhein on A549, A549/Taxol, PC9, and PC9/GD, cell viability was determined by MTT assay. The reduction of cell viability was greater in A549 cells than in A549/Taxol cells after treatment with 0-120 *μ*M Taxol (*P* < 0.05, Figure 1(a)). Similarly, the reduction of cell viability was greater in PC9 cells than in PC9/GD cells after treatment with 0-4 *μ*M gefitinib (*P* < 0.05, Figure 1(b)). The effect of rhein on cell viability was dose-dependent in the range of 5-100 *μ*g/ml, while there was no significant difference between A549 and A549/Taxol in the range of 5-50 *μ*g/ml. And there was a similar effect between PC9 and PC9/GD (*P* < 0.05, Figure 1(c)). The IC50 values for rhein in each cell lines are 47.3 *μ*g/ml (A549), 48.6 *μ*g/ml (A549/Taxol), 24.9 *μ*g/ml (PC9), and 27.4 *μ*g/ml (PC9/GD). These results suggest that rhein is potent in suppressing cell proliferation in both chemosensitive and chemoresistant lung cancer cells.

### 3.2. Rhein Suppresses Colony Formation in Chemosensitive and Chemoresistant Lung Cancer Cells

**Figure 2:**
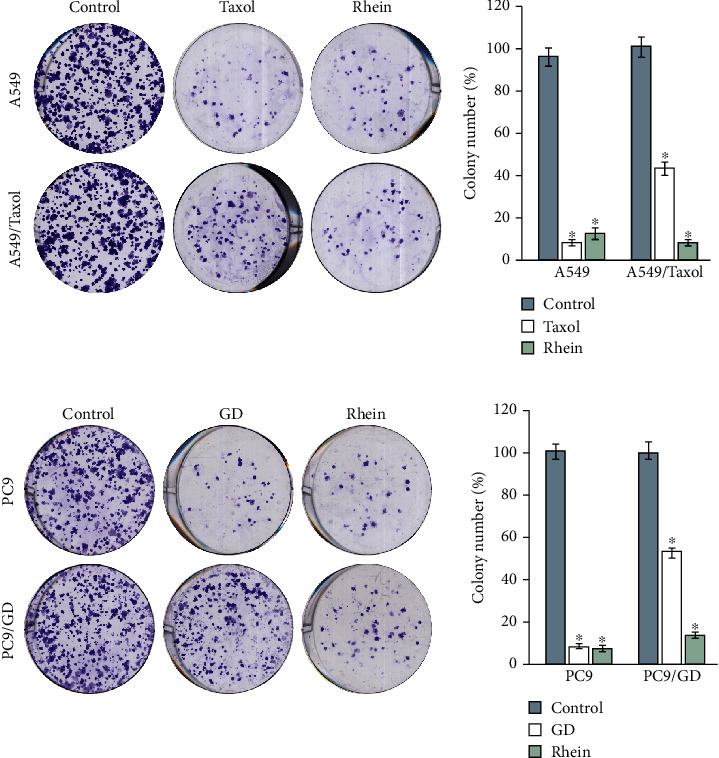
Rhein suppresses colony formation in chemosensitive and chemoresistant lung cancer cells. Images are representative of three independent experiments. Bar graph representing the statistical results of colony number. Data are expressed as the mean ± standard deviation (error bars) from at least three independent experiments. ^∗^*P* < 0.05.

Colony formation is a common method to study the survival and proliferation of adhesive cells. In the current study, the colonies in A549/Taxol and PC9/GD were more than those in A549 and PC9 after Taxol or GD treatment, respectively. In response to treatment with rhein, the colonies in all the cells were significantly decreased, particularly in A549 and PC9 (Figures 2(a) and 2(b)).

### 3.3. Rhein Suppresses the Metastasis of Chemosensitive and Chemoresistant Lung Cancer Cells

**Figure 3:**
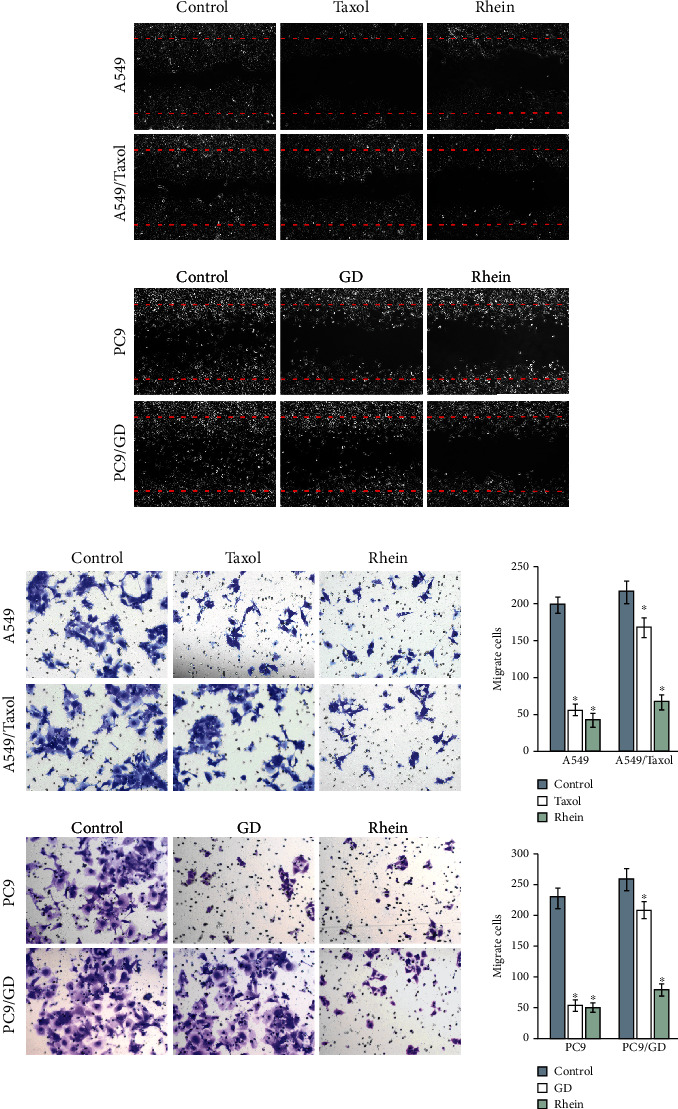
Rhein suppresses the metastasis of chemosensitive and chemoresistant lung cancer cells. (a) The migration of lung cancer cells. (b) Cells migrate to the lower side of the Transwell membrane under a microscope. ^∗^*P* < 0.05 compared to the control group.

The results revealed that rhein reduced the migration of A549, A549/Taxol, PC9, and PC9/GD cells (Figure 3(a)). Compared with the control group, rRhein-treated cells have lowered the wound closure rates (P<0.05). Moreover, rhein also inhibited the migration of lung cancer cells (Figure 3(b)). Overall, rhein can suppress the metastasis of chemosensitive and chemoresistant lung cancer cells.

### 3.4. Rhein Regulated Expression of MMP2, MMP9, Snail, and Stat3

**Figure 4:**
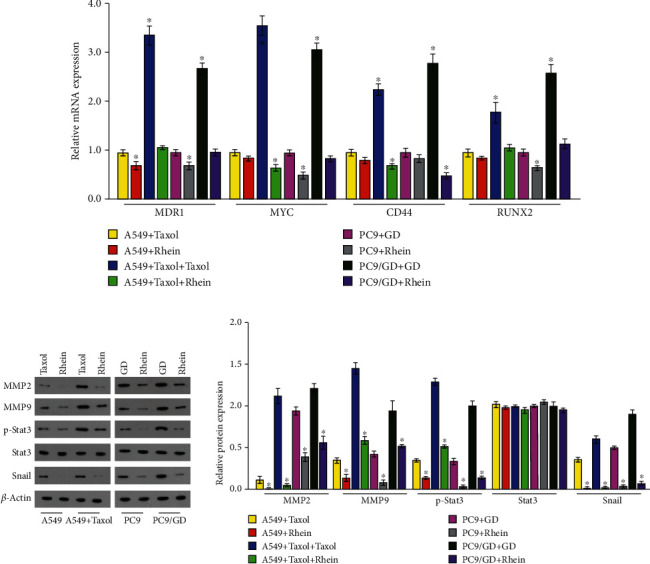
Rhein regulated expression of MMP2, MMP9, Snail, and Stat3. A549, A549/Taxol, PC9, and PC9/GD cells were stimulated with rhein (0 *μ*g/ml or 25 *μ*g/ml or 50 *μ*g/ml) for 24 h. (a) The mRNA levels of MMP2, MMP9, and Snail in A549, A549/Taxol, PC9, and PC9/GD cells were detected by real-time PCR. (b) The levels of MMP2, MMP9, Snail, and Stat3 and phosphorylation of Stat3 in A549, A549/Taxol, PC9, and PC9/GD cells were detected by western blotting. ^∗^*P* < 0.05.

To further explore the mechanism whereby rhein suppressed the migration in lung cancer cells, A549, A549/Taxol, PC9, and PC9/GD cells were treated with 25 *μ*g/ml or 50 *μ*g/ml rhein for 24 h. Then, real-time PCR and western blotting were performed. The results showed that the mRNA and protein levels of MMP2, MMP9, and Snail were decreased significantly, along with the inhibited activation of Stat3. Overall, in lung cancer cells, the Stat3 signaling pathway is the target, whereby rhein suppresses migration (Figures 4(a) and 4(b)).

## 4. Discussion

Lung cancer remains the leading cause of cancer-related deaths worldwide [1]. Chemotherapy is a systematic treatment used to control primary and metastatic lung cancer. But as resistance to various chemotherapeutic drugs has emerged, the response rate to chemotherapy has dropped to only 20-35% [30]. In order to improve the effectiveness of treatment, previous study pays more attention on multitarget therapy, where most traditional Chinese medicines and monomer, including rhein, have their advantages. In recent years, studies on natural herb and their monomers that can inhibit or reverse the occurrence and metastasis of tumors have received extensive attention.

Rhein is a lipophilic anthraquinone, which is widely found in Chinese traditional medicines such as rhubarb, cassia seed, and Polygonum multiflorum [31]. Rhein has been shown to have antitumor effects in a variety of tumors. For example, Zhuang et al. [16] reported that rhein sensitizes human colorectal cancer (CRC) cells. When combined with EGFR-TKIs, rhein may be a novel STAT3 inhibitor in CRC. Wu et al. [32] found that rhein could reverse drug resistance in SMMC-7721/DOX (doxorubicin-resistant cells) cells by inhibiting energy metabolism and inducing mPTP opening. In the present study, we made clear that rhein could suppress cell proliferation and migration in both the chemosensitive and chemoresistance lung cancer cells with MTT assay, colony formation, wound healing assays, and Transwell migration assay.

Lung cancer metastasis involves the regulation of multiple signaling pathways [33]. Numerous studies have shown that STAT3 is involved in multiple metastasis steps and plays an important role in the metastasis of different tumors, including lung cancer [34]. Stat3 is tightly regulated by phosphorylation and transmits signals through multiple signal transduction pathways, such as activation of target genes that mediate metastasis and proliferation [25]. Snail is one of the transcription repressors which can regulate the EMT process. The metastasis of cancer cells depends in part on the EMT process and matrix metalloproteinases, such as Snail, MMP2 and MMP9. According to the results of real-time PCR and western blotting, rhein inhibited the phosphorylation of Stat3, while the transcription of Snail, MMP2, and MMP9 was inhibited by the downregulation of phosphorylation of Stat3. These data explain the mechanism by which rhein inhibits metastasis, in which the STAT3 pathway is regulated by rhein and plays an indispensable role in this process. These observations suggest that rhein may provide new therapeutic options for the prevention of lung cancer progression and metastasis.

## 5. Conclusions

In conclusion, we found that rhein inhibits cell proliferation and migration through the Stat3/Snail/MMP2/MMP9 pathway in lung cancer cells. Thus, rhein may be a potential drug and the Stat3/Snail/MMP2/MMP9 pathway may represent novel potential targets for the treatment of lung cancer. However, the regulatory effects and mechanisms of rhein in other signaling pathways remain to be further studied.

## Data Availability

All data included in this study are available upon request by contact with the corresponding author.
